# Kyste synovial de la bourse retro achilléenne: localisation rare

**DOI:** 10.11604/pamj.2021.38.160.28135

**Published:** 2021-02-12

**Authors:** Hicham Douma, El Hassani Abdelkrim

**Affiliations:** 1 Department of Traumatology, Faculty of Medicine and Pharmacy of Marrakech, Avicenne Military Hospital, University Cadi Ayyad of Marrakech, Marrakech, Morocco,; 2 Department of Rheumatology, Faculty of Medicine and Pharmacy of Marrakech, Avicenne Military Hospital, University Cadi Ayyad of Marrakech, Marrakech, Morocco

**Keywords:** Kyste synovial, radiographie, maladie de Haglund, Synovial cyst, X-ray, Haglund's deformity

## Abstract

Synovial cyst of the retroachilles bursa is a cystic formation developing from the retroachilles bursa. It is a rare cause of talalgia which has been very little reported in the literature. We report the case of a 22-year-old woman suffering from rebel left talalgia resistant to medical treatment. Left foot X-ray showed sharp and protruding bony outgrowth above and behind the great calcanean tuberosity. Chauveaux-Liet angle was 16 degrees and the Fowler-Philip angle was 82 degrees. The diagnosis of Haglund's deformity was retained. Dorsal surgery was performed. The cyst of the retroachilles bursa was removed and sent to the laboratory for anatomopathological examination. This confirmed the diagnosis of synovial cyst. The surgical gesture was complemented by removal of bony outgrowth. Patient's clinical outcome was favorable. This study reports a rare and unusual case of synovial cyst in an extra-articular location, complicating Haglund's deformity.

## Image en médecine

Le kyste synovial rétro-achilléen est une formation kystique développée à partir de la bourse rétro-achilléenne. Il représente une étiologie rare de talalgies. Il est très peu rapporté dans la littérature. Nous rapportons le cas d'une jeune femme de 22 ans, souffrant de talalgies gauches rebelles au traitement médical. La radiographie de profil du pied gauche a montré une excroissance osseuse pointue et saillante en haut et en arrière de la grosse tubérosité calcanéenne. L'angle de Chauvaux était à 16 degrés et l'angle de Fowler-Philip était à 82 degrés. Le diagnostic de la maladie de Haglund a été retenu. Au bloc opératoire, l'abord chirurgical était fait par voie dorsale. Une formation kystique retro-achilléenne a été retrouvée et envoyée pour examen anatomo-pathologique. Ce dernier a confirmé la nature de la formation: un kyste synovial. Le geste a été complété par une résection de l'excroissance osseuse. L'évolution clinique était favorable. Notre cas est original car il montre une localisation rare et inhabituelle d'un kyste synovial en extra-articulaire, compliquant la maladie de Haglund.

**Figure 1:**
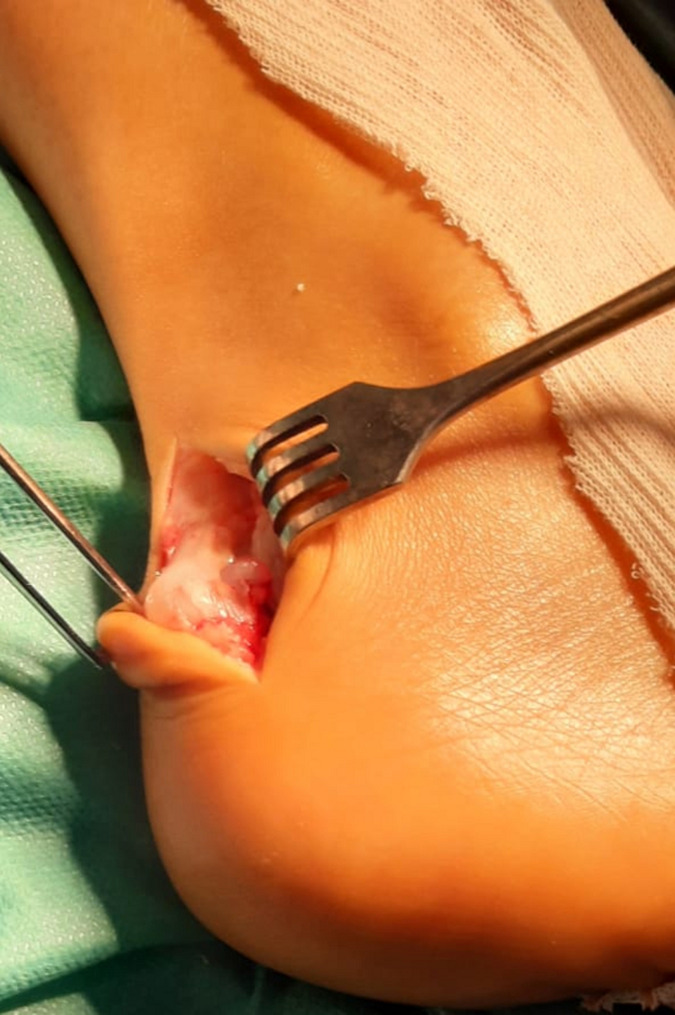
image peropératoire montrant le kyste synovial retro-achilléen

